# Coexistence of hereditary spherocytosis with *SPTB P.Trp1150* gene variant and Gilbert syndrome: A case report and literature review

**DOI:** 10.1515/biol-2022-0904

**Published:** 2024-06-27

**Authors:** Changwei Chi, Shenghao Wu, Wenjin Zhou, Yingying Hu, Yanwei Lu, Shanshan Weng

**Affiliations:** Department of Hematology, The Dingli Clinical College of Wenzhou Medical University (The Second Affiliated Hospital of Shanghai University, Wenzhou Central Hospital), Wenzhou, Zhejiang, China

**Keywords:** hereditary spherocytosis, Gilbert syndrome, jaundice

## Abstract

A congenital protein anomaly in the erythrocyte membrane skeleton causes a hereditary haemolytic illness known as hereditary spherocytosis (HS). The primary characteristic of HS is an increase in the number of tiny spherical red blood cells in the peripheral blood. The chief clinical features of HS include anaemia, jaundice, splenomegaly, spherical erythrocytosis in the blood, chronic anaemia with haemolysis, and recurrent acute attacks. Most patients have a family history; some have autosomal recessive inheritance, whereas most have autosomal dominant inheritance. In cases of severe hyperbilirubinemia disproportionate to haemolysis, other causes of hyperbilirubinemia should be considered. Gilbert syndrome (GS) is an autosomal dominant illness caused by the reduced activity of uridine diphosphate-glucuronosyl transferase lAl and is characterised by intermittent hyperbilirubinemia without any other signs or symptoms of liver disease. The possibility of the coexistence of HS and GS is very limited. Here we present the case of an elderly man with yellow skin and sclera recurring anaemia, and a final diagnosis of coexisting HS and GS.

## 1 Background

Hereditary spherocytosis (HS) and Gilbert syndrome (GS) are autosomal dominant and recessive hereditary diseases, respectively. The probability of the simultaneous occurrence of these two hereditary diseases is extremely low, and its clinical manifestations is jaundice with elevated unconjugated bilirubin levels, which makes its diagnosis difficult.

GS is characterised by intermittent hyperbilirubinemia without liver disease or haemolysis. In patients with GS, the activity of uridine diphosphate-glucuronosyl transferase lAl (UGT1A1) decreased to less than 30% of that in normal individuals [1]. HS is the most common cause of congenital haemolytic anaemia, which leads to premature cell destruction due to erythrocyte membrane defects. Common clinical manifestations include anaemia, jaundice, and varying degrees of splenomegaly. *SPTB*, *ANK1,* and *SLC4A1* variants are common in autosomal dominant HS, whereas new *SPTB* and *ANK1* variants are more common in autosomal recessive HS. Thus, research on HS poses considerable challenges [2]. A unique HS variant spectrum exists in the Chinese population, with *SPTB* variants accounting for 45%, *ANK1* variants accounting for 45%, and *SLC4A1* variants accounting for 10% of case [3]. All variants were non-reproducible, but each was unique to a family member and specific to most Chinese populations. In some patients, multiple variants of related genes can produce synergistic or inhibitory effects, resulting in a complex HS pathogenesis. If HS is comorbid with GS, it can lead to obvious inconsistencies between the genotypes and phenotypes, leading to missed diagnoses and misdiagnoses of clinical HS. Here we present a case report of a patient with HS who harboured a heterozygous variant of the SPTB gene c.3449G＞A (p.Trp1150*) complicated by GS. After treatment, the symptoms of anaemia improved, whereas those of severe jaundice persisted. Finally, the diagnosis was confirmed by combining the clinical findings, laboratory examination results, and molecular sequencing of *UGT1A1* with the hereditary polycythaemia genome set.

## 2 Case presentation

A 70-year-old man was admitted to our department for “dizziness and fatigue since 1 year, which were exacerbated with yellowing of the skin and sclera since 2 months.” One year prior, the patient developed dizziness and fatigue without any obvious cause that was not taken seriously. Two months prior, he experienced dizziness and fatigue, yellowing of the skin and sclera, and deepening of the urine colour. He was treated in other hospitals, and the relevant examination results were as follows: total bilirubin level, 91 μmol/L; direct bilirubin level, 12 μmol/L; indirect bilirubin level, 79 μmol/L; white blood cell count, 9.85 × 10^9^/L; haemoglobin (Hb), 103 g/L; and platelet count, 318 × 10^9^/L. A whole blood examination to identify abnormal red blood cell morphology revealed multiple stained red blood cells, a few large red blood cells, approximately 0.4% broken red blood cells, and approximately 60 spherical red blood cells. The serum-free Hb level was 7.4 mg/dL. The provisional diagnosis was HS and the patient was treated to promote red blood cell production, protect the liver, and eliminate the jaundice.

The patient was discharged after symptom improvement. At that time, the results of the genetic examination were not reported, and the diagnosis was unclear. After discharge, the patient’s symptoms recurred and became exacerbated. The patient was then referred to our hospital for treatment. A physical examination on admission revealed the following: stable vital signs, anaemia, yellow skin, and sclera, no liver palms or spider naevi, no cardiac or pulmonary abnormalities, a soft abdomen, no palpable liver or spleen under the ribs, negative Murphy’s sign, negative McBurney’s point tenderness, normal bowel sounds, negative mobile dullness, and no oedema in either lower limb. The pathological examination showed the following: white blood cell count, 4.3 × 10^9^/L; Hb level, 68 g/L; red blood cell count, 1.75 × 10^12^/L; haematocrit, 21.4%; mean Hb content, 38.9 pg; mean corpuscular volume, 122.3 fL; red blood cell distribution width, 24.7%; platelet count, 366 × 10^9^/L; and reticulocyte percentage, 16.0%. A peripheral blood smear examination revealed visible multicoloured and spherical red blood cells, with approximately 11.5% spherical red blood cells.

**Figure 1:**
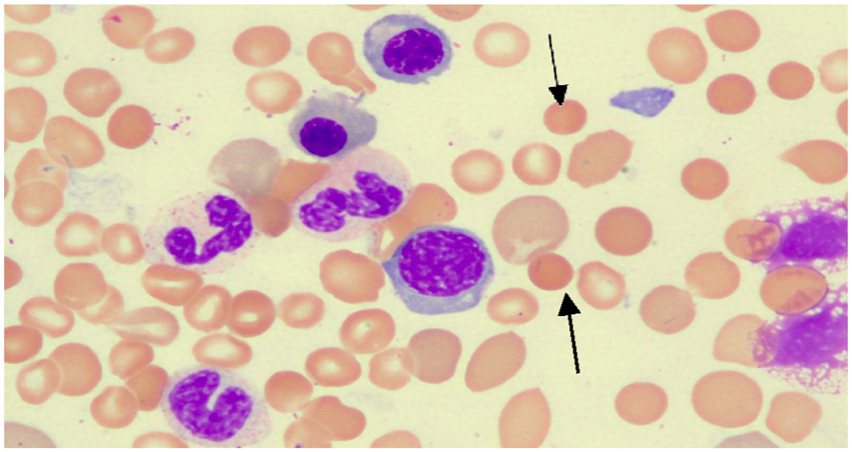
Peripheral blood smear (arrows show small spherical red blood cells) (HE staining).

Liver function tests revealed the following: normal transaminase; total bilirubin level, 145.2 μmol/L; direct bilirubin level, 17.8 μmol/L; indirect bilirubin level, 127.4 μmol/L; lactate dehydrogenase level, 446 U/L; and urine iron-containing Hb, positive (+). The ferritin level was 635.9 μg/L, while the folate, vitamin B12, and glucose-6-phosphate dehydrogenase levels were normal. The antinuclear antibody spectrum test, Coombs direct and indirect tests, the paroxysmal nocturnal haemoglobinuria detection, the cold agglutinin test, and haemoglobin electrophoresis results were normal. Whole abdominal computed tomography revealed a possible left-lobe cyst in the liver and an enlarged spleen. A routine bone marrow examination showed a significant increase in the number of nucleated cells (*G* = 33.5%, *E* = 57.5%; granulocytes:erythrocytes = 0.6:1). Erythroid proliferation was significantly increased, with visible binucleated, enlarged erythroid cells, and Howell–Jolly bodies. Mature red blood cells of various sizes, polychromatic erythrocytes, and spherical red blood cells were also observed (Figure 1). Bone marrow flow cytometry and biopsies revealed no abnormalities. A bone marrow chromosomal examination revealed 47, XY, +8[20], an acquired genetic variation.

**Figure 2:**
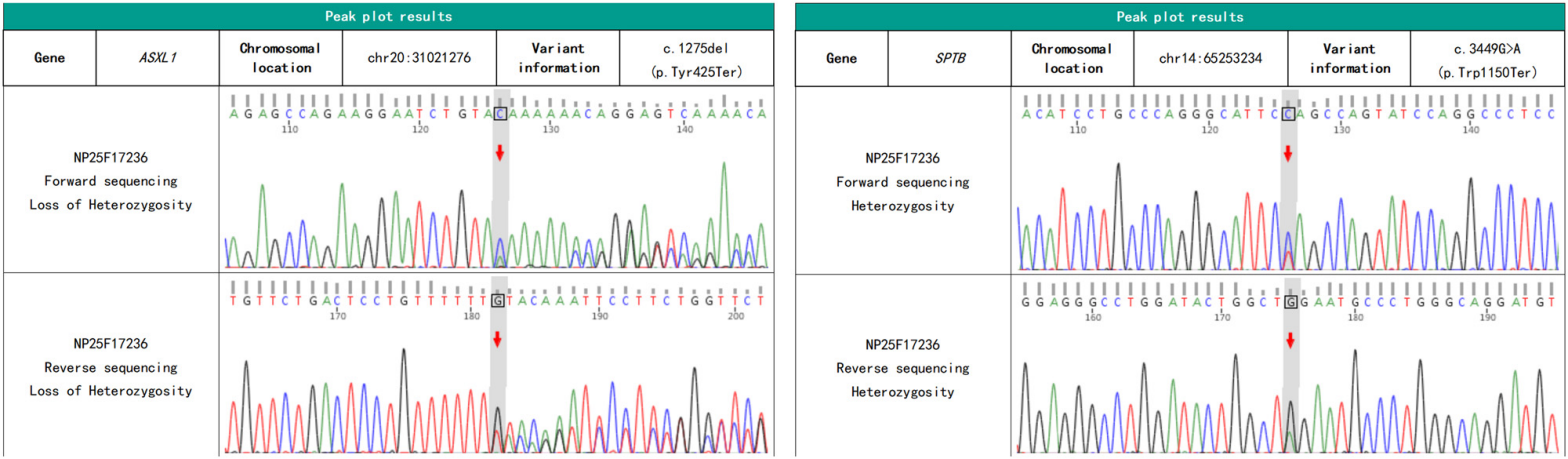
Sanger sequencing results of *SPTB* and *ASXL1* genes (the overlapping peaks caused by insertion mutation in the sequencing signal during unidirectional sequencing precisely indicate that a base is missing here).

**Figure 3:**
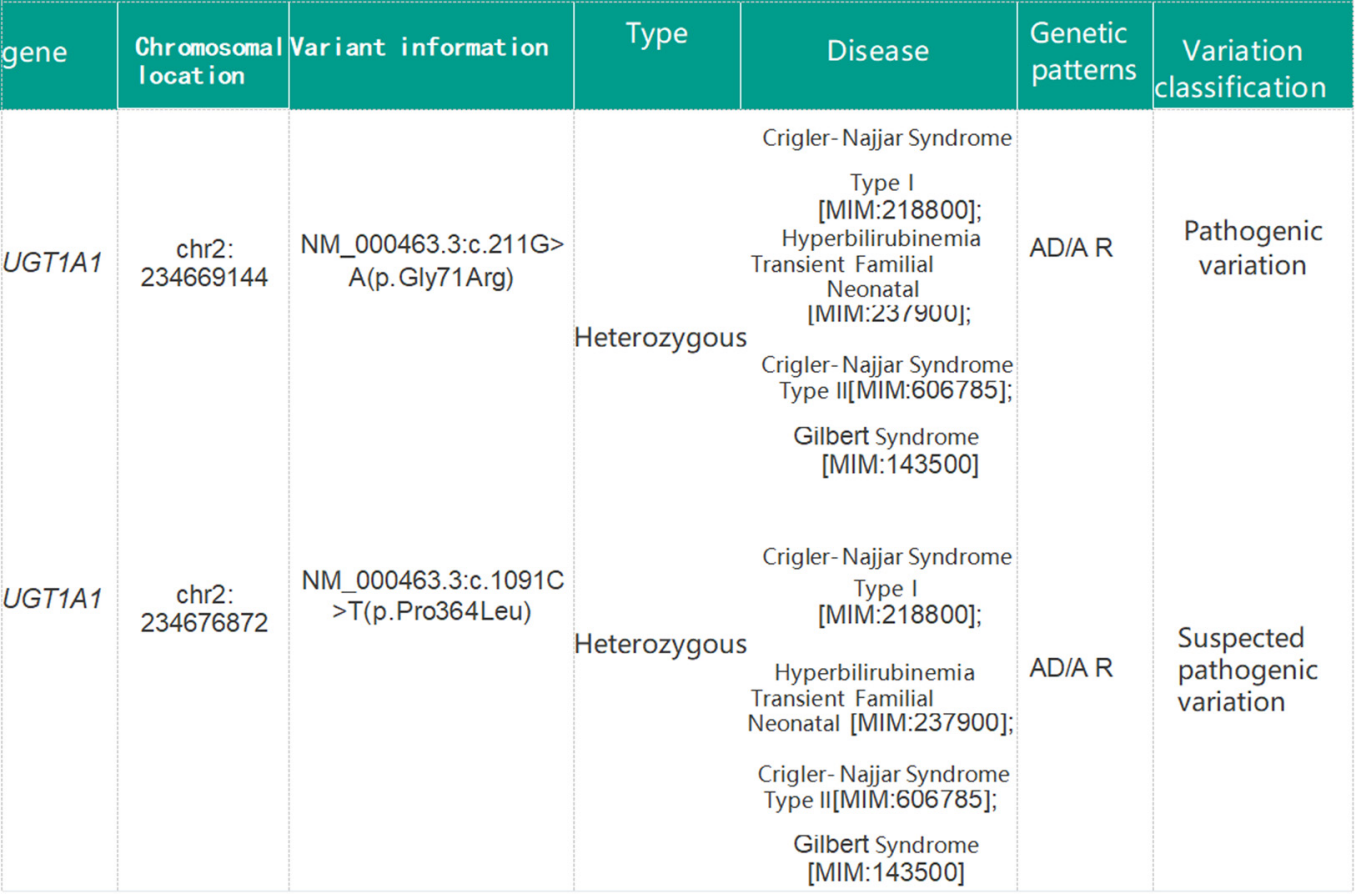
Sequencing results of *UGT1A1* gene.


Considering the possibility of HS and myelodysplastic syndrome, haemolytic anaemia was diagnosed. After treatment, routine blood test results showed an Hb level of 94 g/L and an indirect bilirubin level of 101.7 μmol/L. After discontinuing the glucocorticoids, the patient continued treatment for liver protection and jaundice reduction. Genetic disease related gene sequencing of the patient’s genomic DNA using peripheral blood samples obtained at another hospital (Figures 2 and 3) showed SPTB c.3449G > A (p.Trp1150*) heterozygosity, ASXL1 c. 1275del (p.Tyr425*) suspected heterozygosity, a Chr8 duplication, *UGT1A1 c. 211G > A(p.Gly71Arg)* heterozygosity, and *UGT1A1 c. 1091C > T (p.pro364Leu)* heterozygosity. Based on the patient’s medical history and all case data, he was diagnosed with HS combined with GS. After discharge, the patient’s indirect bilirubin level decreased to 87 μmol/L after rest and liver protection therapy, and he is currently undergoing follow-up in the outpatient clinic.

**Informed consent**: Informed consent has been obtained from all individuals included in this study.
**Ethical approval**: The research related to human use has been complied with all the relevant national regulations, institutional policies and in accordance with the tenets of the Helsinki Declaration, and has been approved by the authors’ institutional review board or equivalent committee.

## 3 Discussion

HS is mostly inherited in an autosomal dominant manner, with some cases being inherited in an autosomal recessive manner. This is caused by variants in the genes located on chromosomes 6 or 8. Owing to membrane defects in the red blood cells, they become spherical, and are destroyed in large quantities when passing through the spleen, resulting in haemolytic jaundice characterised by increased indirect bilirubin levels. Typical clinical manifestations include anaemia, jaundice, and splenomegaly, with some patients having gallstones [4]. The incidence rate in this population is approximately 1/2,000.

HS exhibits significant heterogeneity in its genetics, molecular genetics, biochemical phenotypes, and clinical manifestations. Approximately 25% of patients have no family history and their conditions are associated with gene variants, phenotypic variations, or autosomal recessive inheritance [5]. HS is caused by variants in genes encoding erythrocyte membrane and cytoskeletal proteins. Five genes associated with HS, including *SPTA1, SPTB, ANK1, SLC4A1,* and *EPB42*. HS is caused by at least one variants of an HS-related gene [6]. These variants reduce the levels of proteins that link the endomembrane skeleton of red blood cells to the outer layer of the lipid bilayer, resulting in the formation of microvesicles in the red blood cell membrane and the gradual spherical transformation of the red blood cells. Spherocytes are susceptible to haemolysis through reduced erythrocyte deformability and phagocytosis by splenic macrophages, and the loss of spectrin appears to be particularly associated with haemolysis severity [7].

The updated HS diagnostic process guidelines for 2021 suggest that a preliminary diagnosis can be made based on typical clinical manifestations and laboratory test findings, such as peripheral reticulocytosis, increased unconjugated bilirubin level, and increased microspherocytic red blood cell counts. The diagnosis can be confirmed if a child has a definite family history. If the child’s family history is negative, relevant screenings, such as the acidified glycerol lysis test, red blood cell osmotic fragility test, and genetic testing, can be performed to assist in the diagnosis. The eosin-5-maleimide binding test is currently recognised as the most convenient, highly sensitive, specific diagnostic method and new guidelines have listed it as a new diagnostic approach [8]. Asymptomatic carriers and patients with mild HS do not require special treatment, whereas patients with moderate to severe HS require a total or partial splenectomy and/or splenic artery embolisation [9].

Our patient had no family history of HS. A preliminary diagnosis of HS was made based on clinical manifestations and laboratory test findings. Subsequently, genetic tests performed at an external hospital revealed SPTB c.3449G > A (p.Trp1150*) heterozygosity, suspected ASXLI c. 1275del (p.Tyr425*) heterozygosity, and Chr8 duplication. We know that *c.3449G > A* (p.Trp1150*) is a nonsense variant in the coding region of the *SPTB* gene, which can theoretically lead to the loss of normal protein function through nonsense-mediated mRNA degradation or premature termination of the encoded amino acid sequence. This variant was not reported previously in the gnomAD. In some patients, multiple variants in related genes can produce synergistic or inhibitory effects, leading to a complex HS pathology. Based on the available evidence, we excluded other diseases of the blood system and finally considered the diagnosis of HS. Moreover, the patient experienced disease onset at an old age, and no similar condition was reported in his immediate family; therefore, we identified a high possibility of *SPTB*-acquired variants.

GS, a benign, familial disorder of bilirubin metabolism that often occurs in adolescents and adults, has a higher incidence in men. Because of the reduced *UGT1A1* activity, the ability of liver cells to process Ibil decreases, causing congenital non-haemolytic jaundice characterised by elevated Ibil levels. The primary clinical manifestation is chronic, intermittent hyperbilirubinemia, without haemolytic or liver disease symptoms [10,11]. Its reported prevalence is 2–5%. GS, which is usually undetectable, can be exacerbated by alcohol consumption, fatigue, infection, trauma, pregnancy, or other triggers. The total serum bilirubin level of patients with GS is usually <3 mg/dL, and the condition is mild with a good prognosis. Treatment is generally not necessary, and any symptoms can be alleviated by avoiding triggers. Phenobarbital treatment is reportedly effective.

GS and HS are genetic diseases caused by different gene variants, and their co-occurrence is low. According to international data for GS and HS, the theoretical incidence rate of the comorbid conditions is 15–35 per 100,000 people [1,12]; however, the actual incidence rate statistics have not been reported. Only a few cases have been reported in China, an incidence that is lower than the theoretical incidence rates in other countries. In addition to being related to race, this could be related to missed diagnoses because of insufficient clinical knowledge. The genetic variant sites of the two genetic diseases differ, and the specific mechanism of their comorbidity remains unclear. There is no evidence that these two proteins have a specific biological relevance [13,14]. GS and HS can co-occur during infancy or old age [15]. When the two conditions coexist, patients first experience a mismatch between the degrees of bilirubinaemia and anaemia; some patients with HS have relatively mild anaemia but high bilirubin levels. Therefore, special attention should be paid to the coexistence of these two diseases. Patients with GS show no signs of extravascular haemolysis such as splenomegaly. In patients with HS, if the characteristics of jaundice become significantly aggravated after exertion and significantly alleviated after rest and hyperbilirubinemia is not relieved after treatment, the possibility of comorbid GS should be considered.

Our patient was diagnosed with late-onset HS and GS and had no relevant family history. The diagnosis of HS could explain his splenomegaly, anaemia, and elevated reticulocyte levels. The increased reticulocyte proportion suggested haemolysis. After hormone treatment, the anaemia improved but the jaundice remained severe, and the degree of haemolysis did not match the increase in total and indirect bilirubin levels. Haemolytic jaundice caused by HS alone does not provide adequate justification considering that hyperbilirubinemia is common in cases of *UGT1A1* variants. *UGT1A1* gene screening results reported by another hospital suggested a co-heterozygous variant of *UGT1A1 c. 211G > A (p.Gly71Arg)* and *UGT1A1 c. 1091C > T (p.pro364Leu)*, a widely mutated gene in the Han Chinese population with GS [16].

In summary, the diagnostic process in this case suggests that the diagnosis of hyperbilirubinemia should be combined with the patient’s clinical manifestations and comprehensive screening should be conducted to determine whether a patient has haemolytic disease or *UGT1A1* gene abnormalities. Upon the diagnosis of haemolytic jaundice, the possibility of genetic and acquired haemolytic anaemia should be considered. Upon the diagnosis of *UGT1A1* gene abnormalities, GS and Crigler-Najjar syndrome should be considered. When elevated bilirubin levels do not match the provisional diagnosis, the coexistence of multiple diseases should be considered. As HS and GS are both genetic diseases, a timely and accurate diagnosis can provide guidance for future genetic counselling.
